# Does Culture Mediate the Effect of Promotion/Prevention Regulatory Focus on Subjective Well-Being? Evidence from an Armenian Sample

**DOI:** 10.11621/pir.2021.0307

**Published:** 2021-09-30

**Authors:** Narine G. Khachatryan, Ani K. Grigoryan

**Affiliations:** a Yerevan State University, Yerevan, Armenia

**Keywords:** Subjective wellbeing, promotion and prevention regulatory focus, horizontal/ vertical individualism, horizontal/ vertical collectivism, mediation

## Abstract

**Background:**

Many studies have proven that promotion focus corresponds to the logic of individualistic culture, while prevention focus is characteristic of collectivistic culture. Armenia, as a post-Soviet country, has not been included in cross-cultural studies, since it is not viewed as a typically collectivistic or individualistic society.

**Objective:**

To investigate how promotion and prevention regulatory foci can predict subjective well-being, as conditioned by individualistic–collectivistic cultural orientations within Armenian society, and to reveal the links between regulatory focus and subjective well-being within Armenian culture, considering the effect of personality–culture fit.

**Design:**

We carried out two studies. In Study 1, regression analysis was conducted to reveal how promotion and prevention foci predicted different aspects of subjective well-being. In Study 2, mediation analysis was conducted to reveal how vertical and horizontal collectivism and individualism mediate the linkage between a promotion or prevention focus, and different aspects of subjective well-being.

**Results:**

Regression analysis replicated the findings of other studies, showing that promotion focus has a great predictive role in subjective well-being, while prevention focus neither predicts or obviates different aspects of subjective well-being. Mediation analysis indicated that vertical collectivism had a partially mediating effect on the linkage between promotion and cognitive, emotional, and psychological aspects of subjective well-being. Vertical individualism had a mediating effect on the linkage between prevention and social well-being.

**Conclusion:**

Vertical collectivism is a consistent pattern in people experiencing subjective well-being when they behave in a promotion-based way in different settings in the Armenian cultural context.

## Introduction

Many studies have examined the impact of cultural values on personality and psychological outcomes, such as positive functioning or subjective well-being. These studies usually examined different cultural dimensions as distinct value orientations and revealed differences between countries and/or cultural groups on the individual or societal level. For example, various studies in cross-cultural psychology have found that subjective well-being is culturally conditioned (Diener, Oishi, & Lucas, 2003; Tov, 2018). Other studies have confirmed that various psychological factors determining subjective well-being — such as self-assessment (Diener & Diener, 1995), beliefs and values (Tov & Nai, 2018), and motivation (Csikszentmihalyi & Asakawa, 2016), as well as goal-setting (Oishi & Diener, 2009), and achievement-related behavior (Kurman, Liem, Ivancovsky, Morio, & Lee, 2015) — have different content depending on the culture.

But this dimensional approach does not fully take into account the dynamic nature of the relationship between individuals and their socio-cultural environment. Many researchers have elaborated a multilevel approach for studying the impact of culture on psychological outcomes, particularly on well-being (Fulmer et al., 2019). Oyzerman (2017) suggests that in modern heterogeneous societies, the research methodology of cultural differences between and within cultures should be reviewed, since culture can be operationalized in different ways. Culture can be thought of as the particular practices of a group, as a core theme (such as individualism, collectivism, or honor), and as a situated cognition (Oyzerman, 2017; Oyzerman & Lee, 2008). Oyzerman proposed that studies on individualism vs. collectivism should expand beyond generalizations about Eastern and Western countries, and that results based on specific differences in each culture and subculture could bring new ways of operationalizing “what is culture and how culture matters” (Oyzerman, 2017, p.17).

### Subjective Well-Being, Promotion/Prevention Regulatory Focus, and Cultural Context

Diener (2006) conceptualized subjective well-being (SWB) as the emotional and cognitive evaluations — both positive and negative — that people make about their lives. He defined three elements which measure SWB: an abundance of positive emotions; a lack of negative emotions; and cognitive evaluation of life satisfaction. The *OECD Guidelines on Measuring Subjective Well-Being* (OECD, 2013) recommend including not only overall life satisfaction, but also people’s evaluations of different domains of their lives, as well as the “meaningfulness” or “eudaimonic” aspect of well-being. Based on these main approaches, three aspects of subjective well-being can be distinguished: cognitive, hedonic, and eudaimonic.

Many studies have shown the impact of various personal factors on subjective well-being. For example, according to the theory of self-determination, the satisfaction of his basic needs (such as autonomy, competence, and attachment) contributes to the individual’s sense of well-being in hedonic and eudaimonic ways (Martela & Sheldon, 2019; Ryan & Deci, 2000). The significance of the satisfaction of basic needs for the individual’s sense of well-being is also supported in cross-cultural studies (Linch, 2004). Some studies relating to an individual’s ambitions, goals, and achievements also found that these contribute to the growth of the sense of subjective wellbeing (Emmons, 2003; Kaftan & Freund, 2018). Among personality factors, regulatory focus as a general motivational orientation also has an impact on subjective well-being. Many studies have shown that regulatory focus determines subjective well-being in different settings (Koopmann, Lanaj, Bono, & Campana, 2016; Ouyang, Zhu, Fan, Tan, & Zhong, 2015).

Regulatory focus theory has had a significant role in studies related to the individual’s motivation and behavior. This theory, based on the hedonistic approach of behavioral understanding — *i.e.*, approaching pleasure and avoiding pain — expands the explanatory models of behavioral regulation to include social factors (Higgins, 1997). Higgins (1998) singles out promotion focus and prevention focus as distinct patterns of behavioral regulation. The first is aimed at achievements, accomplishments, desired outcomes, and end-states, while the second aims at avoiding certain outcomes, losses, and end-states of the past.

Referring to the theory of self-discrepancy, Higgins (1998) affirms that promotion and prevention are two different ways of regulating pleasure and pain. In the case of an “ideal self-guide,” the individual has ideas about his/her aims, aspirations, desires, and these are presented as maximal goals; self-congruence is ensured by a positive result, and self-incongruence by the absence of a positive result. In the case of an “ought self-guide,” the individual has ideas about his/her duties, obligations, and responsibilities, which are presented as minimal goals. Here, self-congruence is ensured by the absence of a negative result, and self-incongruence by the presence of one. Other studies also indicate that the promotion or prevention regulatory focus contributes to certain emotions, such as eagerness for promotion focus and vigilance for prevention focus (Higgins et al., 2001), as well as cognitive processes, such as information-processing perception (Hamamura, Meijer, Heine, Kamaya, & Hori, 2009), and making choices and decisions (Zhang & Mittal, 2007).

Regulatory focus also has cross-cultural variability. Cross-cultural studies show that a promotion focus corresponds to the logic of individualistic culture, and a prevention focus to the logic of collectivistic culture (for a review, see Lee & Semin, 2009). In addition, regulatory focus can be a good predictor of cross-cultural differences in achievement-related behavior (Kurman et al., 2015). Some studies identify differences of promotion vs. prevention focus within a culture. Regarding promotion/prevention regulatory focus, Kurman and Hui (2011) show that the division is not absolute, and that these can both be manifested within the same culture, although the authors say that further research is needed in this direction.

## Current Research

The main purpose of this study was to reveal the links between regulatory focus and subjective well-being within Armenian culture, considering the effect of personality– culture fit. Armenia, as a post-Soviet republic with a profound ethnic heritage, has not been included in cross-cultural studies, since it is not viewed as a typically collectivistic or individualistic society. There is a widespread opinion that Armenian society is close to the West in its aims and aspirations, but close to the East in its lifestyle.

Previous findings, though few in number, have shown that Armenian society can be generally characterized as collectivistic. According to the World Values Survey (waves 1997, 2011) (Inglehart et al., 2014) and European Values Study (wave 2008) data analysis, security values are predominant, as opposed to self-expression values. According to the Schwartz Value Survey, the embeddedness value is expressed by Armenians more than the autonomy value on the cultural level. Among the basic 10 value orientations, conformity, benevolence, and security had high ratings on the individual level (Khachatryan, Manusyan, Serobyan, Grigoryan, & Hakobjanyan, 2014). In the same study, the differences between groups and genders showed that for youth and women, the achievement value orientation was salient.

These results showed that, based on value orientations, Armenia can be characterized as having a collectivistic culture, but with a tendency towards individualistic values. In a recent cross-cultural study that compared the individual-level and sample-level predictive utility of a measurement of the cultural patterns of dignity, honor, and face, Armenia was categorized as having an honor culture (Smith et al., 2020). In honor cultures, the acquisition and maintenance of authority for oneself and for one’s group is primary, and this is particularly characteristic of Mediterranean, Latin American, and South Asian cultures (Smith et al., 2017). Honor culture has a different logic than individualistic and collectivistic ones, in which dignity and face cultural logics are more relevant.

The purpose of our study was to investigate how promotion/prevention regulatory focus can predict subjective well-being conditioned by individualistic–collectivistic cultural orientations within Armenian society. We examined the associations between regulatory focus and different aspects of subjective well-being: the cognitive aspect as satisfaction with different life domains, as well as the hedonic and eudaimonic aspects. Two studies were conducted to this end.

## Study 1

The first study was based on the following logic. Promotion is a predictor factor for subjective well-being, and this relation is common for individualistic cultures, while prevention is a more relevant motivational pattern in collectivistic cultures. The question for our study was whether promotion will still predict subjective well-being in Armenian culture, which has features of collectivistic culture and culture of honor. Thus, the hypothesis for Study 1 was the following: *Both promotion and prevention regulatory foci can be predictive factors for subjective well-being.*

## Methods

### Participants

Study 1 used a sample of 223 participants, of whom 107 (48%) were women, with an average age of 19 (*SD* = 1.1702; range 16–22). The participants were students in different disciplines from different universities in Yerevan, Armenia.

### Questionnaires

#### Regulatory Focus Scale (RFS)

An 11-item questionnaire was used to measure the dispositional focus on promotion and prevention (Higgins et al., 2001). Six questions quantify promotion, and five questions quantify prevention. Participants were asked to respond to items on a 5-point Likert-type scale based on the frequency of the specific events in their lives (1–never or seldom; 5–very often). The Armenian version was adapted through the common procedure: translation, back-translation, and comparing with original version. The internal consistency for the promotion subscale was 0.573; for prevention it was 0.678.

#### Personal Well-Being Index (PWI)

This instrument was used to measure the level of satisfaction across eight aspects of personal life — standard of living, health, achievements in life, personal relationships, safety, community connectedness, future security, spirituality/religion, and satisfaction with one’s whole life (International Wellbeing Group, 2013). We measured the cognitive aspect of subjective well-being. The instrument consists of nine items; participants were asked to rate their satisfaction on a Likert scale (from 0 to 10). The Armenian version was adapted through the common procedure: translation, back-translation, and comparing with original version. The internal consistency of the instrument was satisfactory (Cronbach’s *∝* = 0.889).

#### The Short Form of the Mental Health Continuum (MHC-SF)

The MHC-SF is a 14-item self-rating assessment tool that combines the three components of well-being: emotional, social, and psychological (Ke yes, 2009). In the MHC-SF, emotional well-being is represented by three items (happy, interested in life, satisfied); psychological well-being (Ryff, 1989) by six items (self-acceptance, environmental mastery, positive relations with others, personal growth, purpose in life, autonomy); and social well-being by five items (social growth, social coherence, social integration, social contribution, social acceptance). According to Keyes’s model, psychological and social well-being are related to the eudaimonic aspect of well-being (Robitschek & Keyes, 2009).

The Armenian version was adapted through the common procedure: translation, back-translation, items comparison with original version. Participants were asked to respond to the items on a 6-point Likert-type scale based on the experiences they had had over the last month (never, once, or twice, about once a week, 2 or 3 times a week, almost every day, or every day). The internal co nsistency for emotional well-being was 0.750, for social well-being 0.600*,* for psychological well-being 0.801.

Statistical analyses were performed using IBM SPSS Statistics for Windows, Version 23.0.

### Results and Discussion

Descriptive statistics of the main variables are presented in *Table 1.* The skewness and kurtosis for all variables were acceptable (between ±2.0; Tabachnick and Fidell, 2013).

**Table 1:** Means and SDs for all the variables in the study

Variable	M	SD	SK	KU
Personal well–being	7.51	1.552	–.97	1.49
Emotional well–being	4.43	.944	–.81	.29
Social well–being	3.13	.985	.19	–.41
Psychological well–being	4.47	.913	–.87	.56
Promotion	3.61	.599	–.47	.36
Prevention	3.57	.785	–.19	–.65

### Regression Analysis

A stepwise linear regression analysis was performed using personal well-being as the outcome, and promotion and prevention as the predictors. This allowed the examination of whether prevention and promotion predicted personal well-being. A significant regression was found (*F*(1, 218) = 39.861; *p <* 0.0001), with an *R*^2^_adjusted_ of 0.166. Both significantly predicted personal well-being: *B*_promotion_ = 0.929, *t*(218) = 5.593, *p* < 0.0001, *B*_prevention_ = 0.285, *t*(218) = 2.247, *p* = 0.026.

Three stepwise linear regressions were calculated to predict different aspects of well-being based on promotion and prevention. A significant regression was found for the relationship between promotion and emotional well-being (*F*(1, 218) = 26.342, *p* < 0.0001; *B* = 0.523, *p* < 0.0001), with an *R*^2^_adjusted_ of 0.104. Also, significant regression equations were found for the relationships between promotion and social wellbeing (*F*(1, 218) = 11.410, *p* = 0.001; *B* = 0.368, *p* = 0.001), with an *R*^2^_adjusted_ of 0.045, and between promotion and psychological well-being (*F*(1, 218) = 63.837, *p* < 0.0001; *B* = 0.729, *p* < 0.0001) with an *R*^2^_adjusted_ of 0.223.

The regression analysis indicates that promotion regulatory focus contributes to subjective well-being. Moreover, these results are consistent across all aspects of subjective well-being: cognitive, emotional, and eudaimonic. Prevention focus is a predictor only for cognitive evaluation of life satisfaction in different life domains, but with little contribution.

The results of Study 1 replicate those of many other recent studies that show the predictive role of promotion focus in subjective well-being (Koopmann et al., 2016; Ouyang et al., 2015). This means that promotion regulatory focus, such as eagerness to gain rewards and positive outcomes, is consistent in the Armenian cultural context with features of collectivism and honor.

### Study 2

The second study focused on the Armenian cultural context. For capturing this and following the logic of Study 1*,* we included self-reported cultural orientations — the vertical/horizontal aspect of collectivism and individualism — as mediators between promotion/prevention and different aspects of subjective well-being. Following Triandis’s (1996) definition of cultural syndromes and the interpretation of vertical and horizontal dimensions of individualism (VI, HI) and collectivism (VC, HC) by Singelis, Triandis, Bhawuk, and Gelfand (1995), we assumed that the cultural patterns of VC-VI-HC-HI can best define the social belief system and value orientations within heterogeneous societies such as Armenia. Thus, the hypothesis for Study 2 was the following: *Vertical collectivism and individualism will mediate the association between promotion/prevention regulatory foci and subjective well-being.*

## Method

### Participants

Study 2 was based upon a sample of 237 participants, of whom 181 (76.1%) were women, with an average age of 22.77 (*SD* = 6.034; range 17–57).

### Questionnaires

#### General Regulatory Focus Measure (GRFM)

This instrument was used to measure people’s promotion and prevention goals; it comprises a total of 18 items (9 promotion and 9 prevention items) to be answered on a 9-point scale ranging from “not at all true of me” to “very true of me” (Lockwood, Jordan, & Kunda, 2002). In contrast to the items in the RFQ, the items in this questionnaire relate to current attitudes, actions, and habits (e.g., “In general, I am focused on preventing negative events in my life,” or “I typically focus on the success I hope to achieve in the future”). The Armenian version was adapted through the common procedure: translation, back-translation, items comparison with original version. The internal consistencies for both subscales were satisfactory (Cronbach’s *α*_promotion_ *=* 0.829; Cronbach’s *α*_prevention_ *= 0.718).* We used this instrument because the internal consistency for promotion/prevention is satisfactory for comparison with the RFS used in Study 1.

The Personal Well-Being Index (PWI) and the short form of the Mental Health Continuum (MHC-SF) were the same as in Study 1.

#### Individualism and Collectivism Scale

The 16-item instrument was used to measure four dimensions of individualism and collectivism (four items for each dimension) (Triandis & Gelfland, 1998):

- Vertical Collectivism (VC) — seeing the self as part of a collective and being willing to accept hierarchy and inequality within that collective;
- Horizontal Collectivism (HC) — seeing the self as part of a collective, but perceiving all the members of that collective as equal;
- Vertical Individualism (VI) — seeing the self as fully autonomous, but recognizing that inequality will exist among individuals and accepting this inequality;
- Horizontal Individualism (HI) — seeing the self as fully autonomous and believing that equality between individuals is the ideal.

All items were answered on a 9-point scale, ranging from 1 = never or definitely no and 9 = always or definitely yes. The Armenian version was adapted through the common procedure: translation, back-translation, items comparison with original version. The internal consistencies for each subscale were: *α*_VColl_ = 0.669; *α*_HColl_ = 0.713; *α*_VInd_ = 0.711; *α*_Hind_ = 0.740.

## Results and Discussion

The mediation analyses were performed using IBM SPSS Statistics for Windows, Version 23.0, in combination with the PROCESS version 3.5 macro by Andrew F. Hayes (Hayes, 2017). The contribution for each mediator was tested in a parallel format and the mediating effect only went via paths a_i_ and b_i_ through the corresponding mediators. This made it possible to compare the effects of each mediator in the model. The significance of indirect effect was tested by bootstrapping procedures.

The first parallel mediation analysis was performed to assess the mediating role of the four cultural dimensions in the linkages between promotion/prevention and personal well-being (PW).

For the mediation model of promotion to personal well-being, two out of the four mediators were found to significantly contribute to their relationship. The indirect effect (IE) of promotion on personal well-being through vertical collectivism was found to be significant (IE = 0.0698, 95% CI [0.0128, 0.1537]), meaning that the effect of promotion on personal well-being was partially mediated by vertical collectivism. The s ignificant indirect effect of vertical individualism had the opposite sign to that of the total effect (IE = –0.1131, 95% CI [–0.2226, –0.0202]), meaning that vertical individualism was a suppressor (MacKinnon, Krull, & Lockwood, 2000). The relationship between promotion and personal well-being was strengthened by including vertical individualism.

**Figure 1.**
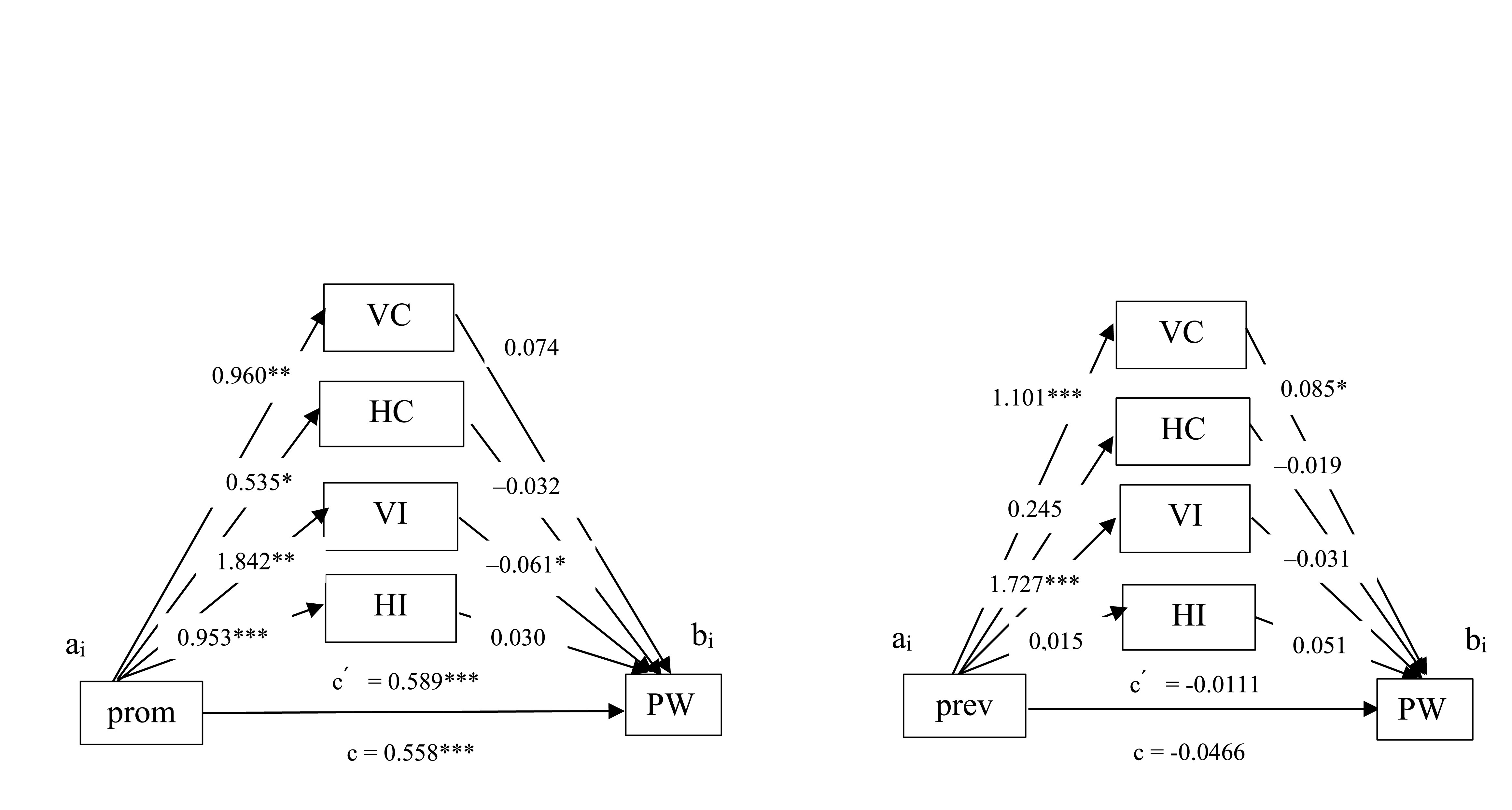
The mediating effect of four cultural dimensions in the relationships between promotion/prevention and personal well-being.

*Note. VC = vertical collectivism; HC = horizontal collectivism; VI = vertical individualism; HI = horizontal individualism; prom = promotion; prev = prevention; PW = personal well-being.*

All effects presented are unstandardized; ai is the effect of promotion/prevention on cultural dimensions; bi is the effect of cultural dimensions on personal well-being; c' is the direct effect of promotion/prevention on personal well-being; c is the total effect of promotion/prevention on personal well-being. *p < .05; **p < .01; ***p < .001.

The mediation analysis indicates that promotion regulatory focus with association to vertical collectivism predicts the cognitive aspect of subjective well-being. We can assume from these results that promotion focus as an achievement-related motivational orientation in the Armenian context can have different manifestations in social behavior. Such differences can be observed in the agency–communion model of narcissism, about which, according to Gebauer and Sedikides (2018), narcissistic, self-empowerment behavior may be manifested in collectivistic cultures; however, the causes of such behavior are connected to the satisfaction not of “self ”-motives, but “we”-motives. Following this logic, we can assume that promotion, as motivation to reach goals and have achievements, has in-group direction and is in accordance with the expectations and opinions of referent people with high status. This kind of hierarchical attachment in relations provides safety, self-enhancement, and maintenance of self-esteem, which have an impact on life satisfaction.

There is no mediation for the model of prevention to personal well-being because the total effect (TE = –0.0111, *p* = 0.9294) and the indirect effect (IE = –0.0466, *p* = 0.7246) were not significant.

The second parallel mediation analysis was performed to assess the mediating role of four cultural dimensions on the linkages between promotion/prevention and emotional well-being (EW).

For t he mediation model of promotion to emotional well-being, two out of the four mediators were found to significantly contribute to their relationship. The indirect effect of promotion on emotional well-being through vertical collectivism was found to be significant (IE = 0.0305, 95% CI [0.0007, 0.0774]), meaning that the effect of promotion on emotional well-being was partially mediated by vertical collectivism. The significant indirect effect of vertical individualism had the opposite sign to that of the total effect (IE = –0.0966, 95% CI [–1.1661, –0.0360]), meaning that vertical individualism was a suppressor. The relationship between promotion and emotional well-being was strengthened by including vertical individualism.

**Figure 2.**
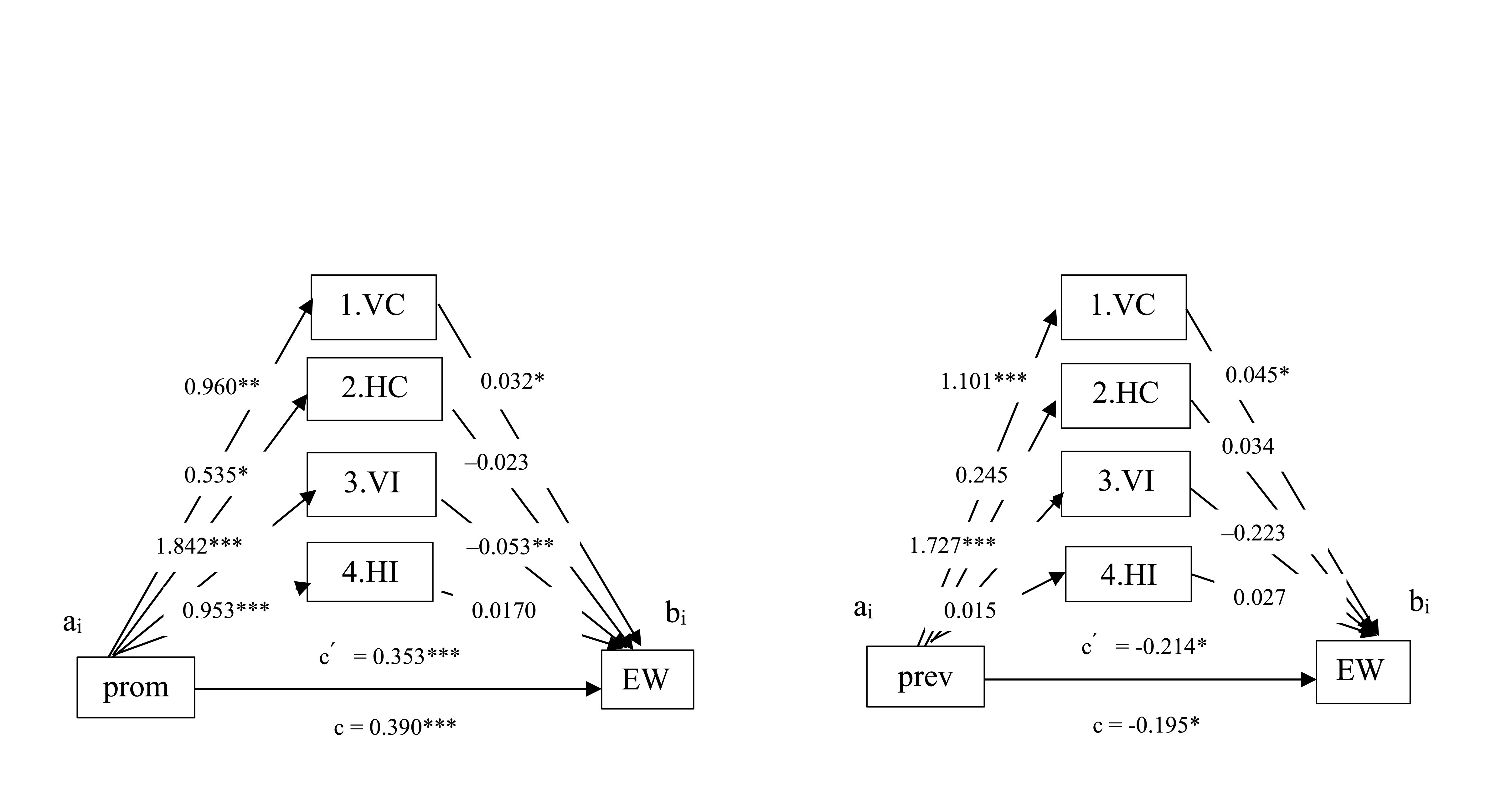
The mediating effect of four cultural dimensions in relationships between promotion/prevention and emotional well-being.

*Note. VC = vertical collectivism; HC = horizontal collectivism; VI = vertical individualism; HI = horizontal individualism; prom = promotion; prev = prevention; EW = emotional well-being.*

*All effects presented are unstandardized; ai is the effect of promotion/prevention on cultural dimensions; bi is the effect of cultural dimensions on emotional well-being; c*' is the direct e! ect of promotion/prevention on emotional well-being; c is the total effect of promotion/prevention on emotional well-being. *p < .05; **p < .01; ***p < .001

The mediation analysis indicates that promotion regulatory focus with an association to vertical collectivism predicts the emotional/hedonic aspect of subjective well-being. These results replicate the previous one, and we can state again that achievements and gaining positive results can raise the sense of subjective well-being when achievements are approved by the referent groups, by members who have high status, thus satisfying the latter’s expectations. This may give a sense of safety, as well as the experience of positive emotions.

For the mediation model of prevention to emotional well-being, one out of the four mediators was found to significantly contribute to their relationship. The significant indirect effect of vertical collectivism had the opposite sign to that of the total effect (IE = 0.0494, 95% CI [0.0113, 0.1003]), meaning that vertical collectivism was a suppressor. The relationship between prevention and emotional well-being was strengthened by including vertical collectivism.

The third parallel mediation analysis was performed to assess the mediating role of four cultural dimensions on the linkages between promotion/prevention and social well-being (SW).

For the mediation model of promotion to social well-being, one out of the four mediators was found to significantly contribute to their relationship. The significant indirect effect of vertical individualism had the opposite sign to that of the total effect (IE = –0.0899, 95% CI [–1.570, –0.0367]), meaning that vertical individualism was a suppressor. The relationship between prevention and social well-being was strengthened by including vertical individualism.

For the mediation model of prevention to social well-being, the indirect effect of vertical individualism was found to be significant (IE = –0.0542, 95% CI [–0.1181, –0.0029), meaning that the effect of prevention on social well-being was completely mediated by vertical individualism. This mediation means that prevention negatively predicts social well-being when the autonomous self is postulated as different from others with the inequality notion. As social well-being is the extent to which people are thriving in their social lives in local and broader communities (Keyes, 1998), the results can make sense, because social aspects of well-being describe satisfaction with one’s prosocial behavior, which is related to the meaningfulness of life.

**Figure 3.**
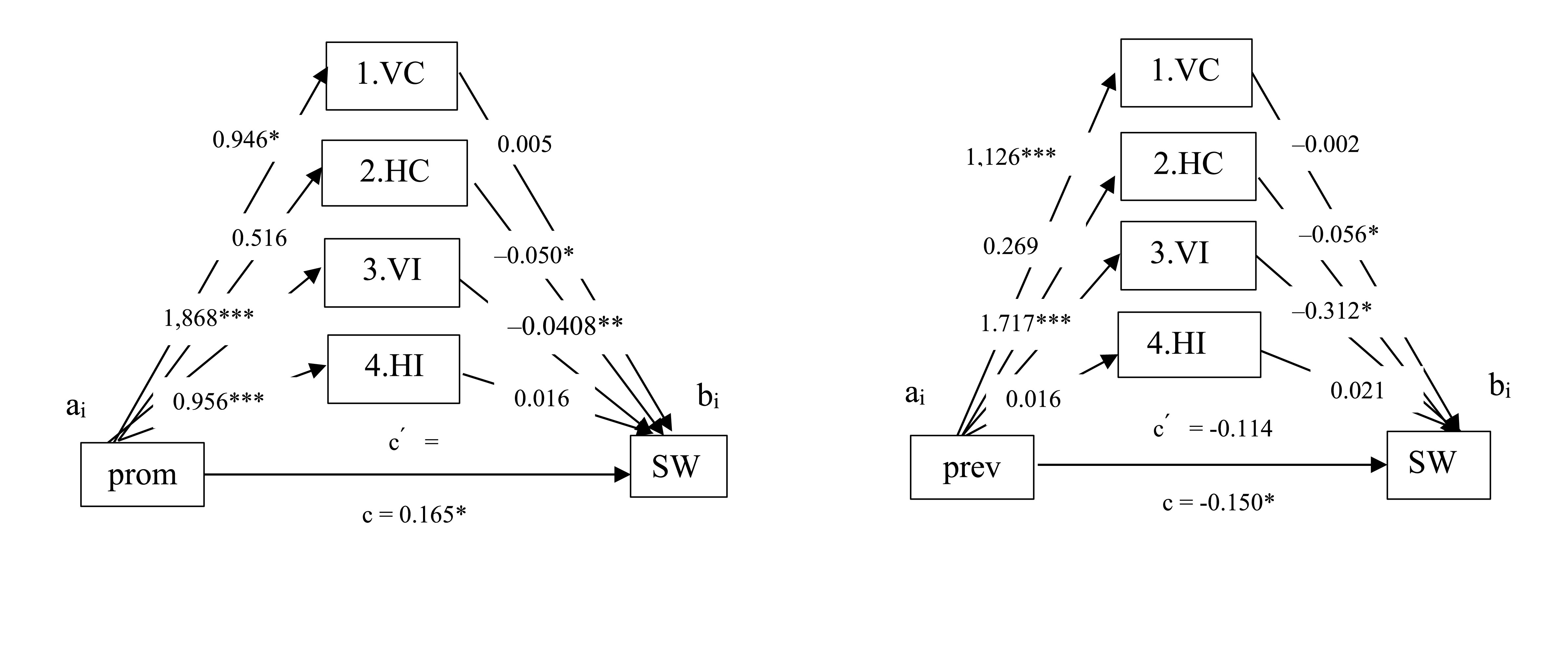
The mediating effect of four cultural dimensions in the relationships between promotion/prevention and social well-being.

*Note. VC = vertical collectivism; HC = horizontal collectivism; VI = vertical individualism; HI = horizontal individualism; prom = promotion; prev = prevention; SW = social well-being.*

*All effects presented are unstandardized; ai is the effect of promotion/prevention on cultural dimensions; bi is the effect of cultural dimensions on social well-being; c*' is the direct effect of promotion/prevention on social well-being; c is the total effect of promotion/prevention on social well-being. *p < .05; **p < .01; ***p < .001.

The fourth parallel mediation analysis was performed to assess the mediating role of al four cultural dimensions on the linkages between promotion/prevention and psychological well-being (PsyW).

For the mediation model of promotion to psychological well-being, three mediators were found to significantly contribute to their relationship. The indirect effects of promotion on psychological well-being through vertical collectivism (IE = 0.0324, 95% CI [0.0007, 0.0789]) and horizontal individualism (IE = 0.0575, 95% CI [0.0175, 0.1138]) were found to be significant, meaning that the effects of promotion on psychological well-being were partially mediated by vertical collectivism and horizontal individualism. The significant indirect effect of vertical individualism had the opposite sign to that of the total effect (IE = -0.636, 95% CI [-0.1295, -0.0085]), meaning that vertical individualism was a suppressor. The relationship between promotion and psychological well-being was weakened by including vertical individualism.

The mediation analysis indicates that promotion regulatory focus with an association to vertical collectivism and horizontal individualism predicts psychological well-being. Psychological well-being is the extent to which people are thriving in their personal lives, for example, in personal growth, self-acceptance, and a sense of purpose in life (Ryff, 1989). Horizontal individualism is a cultural pattern in which an autonomous self is postulated, but individuals perceive themselves and others as equal in status. If horizontal individualism can be a relevant factor for psychological well-being, vertical collectivism is a non-typical cultural pattern in this. Meanwhile, comparing the mediation effects of previous models, we have a replication of the significance of vertical collectivism as a predictive factor for subjective well-being. According to these results, we can assume that different aspects of psychological well-being, such as personal growth, self-acceptance, personal goals, positive relations with others, purpose in life, and mastery can be realized in the context of competitiveness with members of the in-group. As vertical collectivism and horizontal individualism have opposite features as cultural patterns in the sense of an independent vs. interdependent self, as well as equality vs. inequality in social relations, we can assume, based on the interpretation of culture as situated cognition, that there can be a shift from one to another depending on situational cues (Oyzerman & Lee, 2008).

**Figure 4.**
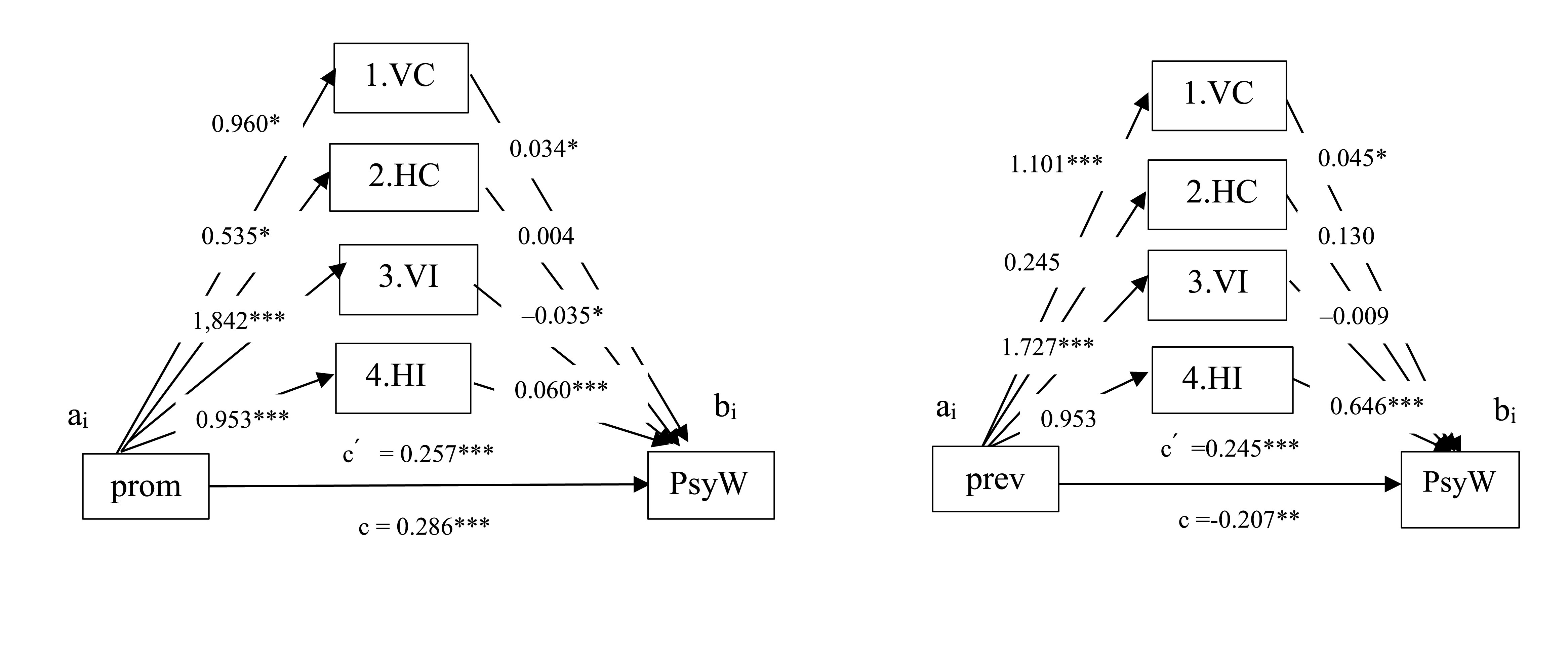
The mediating effect of four cultural dimensions in the relationships between promotion/prevention and psychological well-being.

*Note. VC = vertical collectivism; HC = horizontal collectivism; VI = vertical individualism; HI = horizontal individualism; prom = promotion; prev = prevention; PsyW = psychological well-being.*

*All effects presented are unstandardized; ai is the effect of promotion/prevention on cultural dimensions; bi is the effect of cultural dimensions on psychological well-being; c*' is the direct effect of promotion/prevention on psychological well-being; c is the total effect of promotion/prevention on psychological well-being. *p < .05; **p < .01; ***p < .001.

For the mediation model of prevention to psychological well-being, the significant indirect effect of vertical collectivism had the opposite sign to that of the total effect (IE = 0.0496, 95% CI [0.0101, 0.1013]), meaning that vertical collectivism was a suppressor. The relationship between prevention and psychological well-being was strengthened by including vertical collectivism.

## General Discussion and Conclusion

The results from the Armenian sample replicate the findings of other studies, particularly that promotion focus has a great predictive role in subjective well-being, while prevention focus does not predict (Study 1) or predicts negatively (Study 2) the different aspect of subjective well-being. However, when we capture cultural orientations and their impact on the predictive role of promotion/prevention regulatory focus in subjective well-being, the specific effects are revealed. Vertical collectivism has a partial mediating effect on the linkage between promotion and the cognitive aspects of subjective well-being, such as satisfaction with different life domains, as well as the emotional aspect and psychological well-being. Based on our findings, we can assume that vertical collectivism is a consistent pattern in experiencing subjective well-being when people behave in a promotion-based way in various settings.

This finding is not consistent with cross-cultural studies showing that a promotion regulatory focus corresponds with individualism, but we can assume that our findings prove the effect of personality–culture fit. As we saw from previous findings, embeddedness as a cultural value is more descriptive than autonomy for Armenian society, and honor is a salient cultural value as well (Smith et al., 2020). Conformity, benevolence, and security are also high among Armenian value orientations (Khachatryan et al., 2014). Putting together our previous and present findings, we can conclude that promotion in the Armenian cultural context can have different manifestations than those in an individualistic cultural context. Thus, promotion-based behavior raises satisfaction with life, positive emotions, and meaningfulness through personal, not social, growth even in collectivistic, unequal, and competitive forms of social interactions.

Our study also illustrated that vertical individualism had a mediating effect on the linkage between prevention and social well-being. Vertical individualism, as an independent sense of self with inequality in relations, fully mediated the negative impact of prevention-based behavior on social well-being. Thus, prevention-based behavior decreased the sense of subjective well-being and the meaningfulness of social growth and proactive behavior in individualistic, competitive, and unequal forms of social interactions.

The results show that in Armenian society, we can speak about both universal and cultural patterns of understanding in the sense of subjective well-being, a finding which can be useful for the study of other societies as well. On the other hand, the results also showed that subjective well-being is a multidimensional phenomenon and can have various manifestations, depending on between-culture and within-culture differences.

## Limitations and Future Studies

One limitation of this research is that demographic factors which could reveal differences within Armenian society, such as gender, age, education, material well-being, and urban vs. rural, were not taken into account. Future studies will be aimed at capturing the moderating effect of such demographic factors, which may show subcultural differences of the predictive role of regulatory focus on different aspects of subjective well-being.

Since there is a lack of cross-cultural studies testing the effects of regulatory focus on personality–culture fit, the other limitation of the study is that the evidence that vertical collectivism is a consistent pattern in mediating subjective well-being when people behave in a promotion-based way, might not be very convincing. To test and replicate this finding, cross-cultural studies need to be done to examine the linkage between regulatory focus and different aspects of subjective well-being in countries both similar and unsimilar to the Armenian cultural context. Future efforts will be aimed at working in collaboration with representatives from different countries on this task.

Based on our results, the other direction for future study could be the study of social-cognitive factors that could determine the negative impact of prevention-based behavior on social well-being in individualistic, competitive, and unequal forms of social interaction. We believe that future findings will be applicable for developing culturally sensitive social policies, facilitating different types of prosocial behavior in Armenian society.
